# Oncolytic Virotherapy in Colorectal Cancer: Mechanistic Insights, Enhancer Strategies, and Translational Combinations

**DOI:** 10.3390/cells14242006

**Published:** 2025-12-16

**Authors:** Huda Salameh, Nesha Naseem, Muhammad A. Chattha, Joytish Ramesh, Haneen Ramy, Dasa Cizkova, Peter Kubatka, Dietrich Büsselberg

**Affiliations:** 1 Weill Cornell Medicine—Qatar, Cornell University, Doha P.O. Box 24144, Qatar; hus4003@qatar-med.cornell.edu (H.S.); nen4001@qatar-med.cornell.edu (N.N.); mac4066@qatar-med.cornell.edu (M.A.C.); jor4029@qatar-med.cornell.edu (J.R.); hmr4002@qatar-med.cornell.edu (H.R.); 2 Centre of Experimental and Clinical Regenerative Medicine, Small Animal Clinic, University of Veterinary Medicine and Pharmacy, 041 81 Kosice, Slovakia; dasa.cizkova@uvlf.sk (D.C.); peter.kubatka@uvlf.sk (P.K.)

**Keywords:** oncolytic virus, colorectal cancer, therapy

## Abstract

**Highlights:**

**What are the main findings?**

- Standalone oncolytic viruses induce tumor-selective lysis and remodel the colorectal tumor microenvironment.
- Combinations with immune checkpoint inhibitors consistently enhance tumor control in colorectal cancer.

**What are the implications of the main findings?**

- Enhancer tools further improve viral delivery, selectivity, efficacy, and evasion of antiviral immune responses.
- Synergy with immune checkpoint inhibitors offers an opportunity to overcome therapeutic resistance in microsatellite-stable colorectal cancer.

**Abstract:**

Colorectal cancer (CRC) is one of the leading causes of cancer-related morbidity and mortality worldwide, with most patients, especially those with microsatellite-stable disease, having limited treatment options. Oncolytic viruses (OVs) have emerged as a promising therapeutic modality due to their ability to selectively replicate in malignant cells and mediate antitumor effects through direct oncolysis, immune activation, and modulation of tumor angiogenesis. This review analyzed 101 primary studies that reported the use of OV in CRC. The extracted data, including virus type, study design, model system, mechanistic pathways, and therapeutic strategies, were organized as standalone therapy, combination therapy, or enhancer-based approaches. Across studies, OV monotherapy consistently induced selective tumor cell lysis and, in some models, also exhibited additional immunogenic and anti-angiogenic effects. Combination strategies, particularly those with immune checkpoint inhibitors, demonstrated synergistic activity, enhancing T-cell infiltration, cytokine production, and tumor control even in resistant CRC settings. Enhancer approaches, including mesenchymal stem cell delivery systems and tumor-specific promoters, have improved viral selectivity, tumor penetration, and reduced immune clearance. Despite promising findings, progress is hindered by heterogeneous models and the scarcity of advanced clinical trials. Translation into well-designed clinical studies is now warranted to optimize therapeutic outcomes.

## 1. Introduction

Colorectal cancer (CRC) is one of the most formidable global health challenges, ranking as the third most diagnosed cancer and the second leading cause of cancer-related mortality [1]. In 2020, over 1.9 million new CRC cases and more than 930,000 related deaths were reported, accounting for 10% of all cancer cases worldwide [2]. With continued demographic aging, urbanization, and the adoption of Westernized lifestyles, the CRC burden is projected to exceed 3.2 million new cases and 1.6 million deaths annually by 2040, with a disproportionate rise among younger populations [1,2]. This shift includes a sustained increase in early-onset CRC (EOCRC), with incidence rising in individuals <50 years, implicating diet, obesity, sedentary behavior, and gut microbiome dysbiosis as contributors [3]. Beyond incidence, colorectal cancer accounts for a substantial disability-adjusted life-year (DALY) burden, attributable primarily to delayed diagnosis and limited access to colonoscopy and molecular testing in resource-constrained [4]. Consequently, these evolving epidemiological trends underscore the urgent need for enhanced preventive strategies and therapeutic innovations to reduce CRC-related morbidity and mortality worldwide.

Standard therapeutic strategies for CRC remain surgery for localized disease, complemented by adjuvant chemotherapy and radiotherapy, and targeted therapies for advanced stages. Standard first-line therapy is built on FOLFOX, FOLFIRI, or FOLFOXIRI backbones, augmented with anti-VEGF agents (e.g., bevacizumab) or anti-EGFR antibodies in RAS wild-type, predominantly left-sided tumors; in oligometastatic presentations, metastasectomy and liver-directed interventions are actively considered [5]. More recently, immunotherapies have been expanding as a promising approach, particularly immune checkpoint inhibitors (ICIs), which target the PD-1/PD-L1 and CTLA-4 pathways. Despite these advances, clinical outcomes remain suboptimal. Chemotherapy efficacy is limited by tumor resistance mechanisms, such as drug efflux modifications and overexpression of ATP-binding cassette transporters, resulting in nearly half of patients experiencing recurrence [6]. Additional resistance mechanisms encompass epithelial–mesenchymal transition (EMT), cancer stem-cell niches, rewiring of DNA-damage response pathways, WNT/β-catenin activation, and stromal remodeling within the tumor microenvironment (TME), each of which undermines the durability of cytotoxic and targeted therapies [7]. Moreover, immune checkpoint blockade has demonstrated remarkable results in only a small subset of patients with mismatch repair deficiency (dMMR) or microsatellite instability-high (MSI-H) tumors, leaving the majority of patients with mismatch repair-proficient (pMMR) or microsatellite stable (MSS) CRC with minimal benefit, as efficacy is further impacted by tumor heterogeneity [8]. MSS/pMMR tumors are typically “immune-cold,” characterized by low tumor mutational burden, poor neoantigenicity, exclusionary TGF-β–driven stroma, myeloid-dominant immunosuppression, and defective antigen presentation, which collectively limit ICI responsiveness [9]. For example, the phase II KEYNOTE-016 trial first demonstrated that PD-1 blockade was effective in MSI-H/dMMR metastatic CRC but not in MSS disease, and the subsequent phase III KEYNOTE-177 trial confirmed these findings in a larger cohort, establishing pembrolizumab as the standard first-line therapy for MSI-H/dMMR metastatic CRC [8]. This population represents less than 15% of all CRC patients; thus, over 80% of metastatic CRC patients experience no meaningful response to checkpoint inhibition [10]. The immune-refractory nature of MSS CRC emphasizes the need for novel approaches that can both directly induce tumor cell death and overcome immune resistance [11].

**Figure 1:**
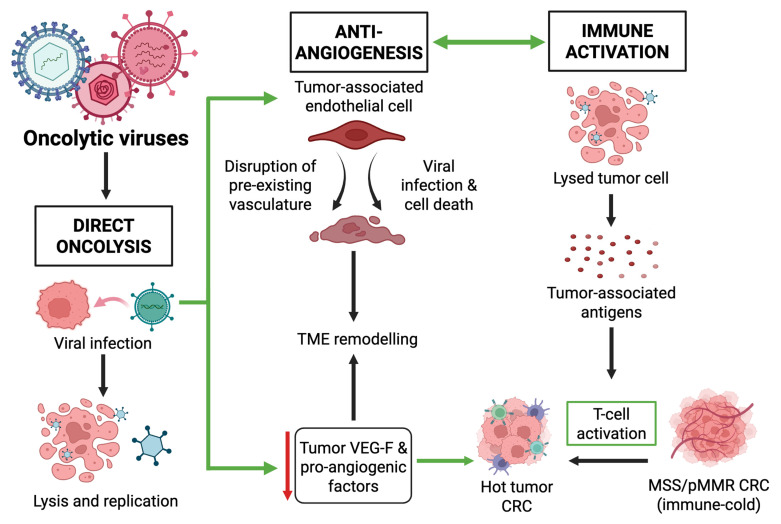
A simplified overview of the main mechanisms of oncolytic virotherapy in CRC. Anti-tumor activity of oncolytic viruses acting on CRC tumor cells is through three core mechanisms: direct oncolysis of infected CRC cells via lysis and replication; immune activation, by which the release of tumor-associated antigens stimulates T-cell activation and subsequent transformation of a ‘cold’ tumor into a ‘hot’ tumor; and anti-angiogenesis, executed through the targeting of tumor-associated endothelial cells and reduced VEGF secretion, thus leading to a TME modulation. These three mechanisms function in a coordinated and interdependent manner during oncolytic virotherapy. Created in BioRender. Busselberg, D. (2025) https://BioRender.com/pq8tsot. Abbreviations: CRC, Colorectal Cancer; MSS/pMMR, Microsatellite Stable/Proficient Mismatch Repair; TME, Tumor Microenvironment; VEGF, Vascular Endothelial Growth Factor. **Green** arrow: interconnected mechanisms, **↓**: decreased.

Oncolytic viruses (OVs) have emerged as a promising therapy in this field. Naturally occurring or genetically engineered viruses selectively replicate within and destroy malignant cells, exerting antitumor effects through several interconnected mechanisms, as shown in Figure 1, while sparing healthy tissue [12]. OVs induce direct tumor cell lysis by disrupting host protein synthesis, driving viral replication until the cell is lysed. This results in the release of replicated viruses, thereby facilitating the spread of infection within the tumor [13]. Mechanistically, OV-triggered tumor cell death is often immunogenic, characterized by the exposure of cell-surface calreticulin and the release of ATP and HMGB1—canonical DAMPs that license dendritic cells for antigen cross-presentation. This process exemplifies how oncolytic virus-induced immunogenic cell death (ICD) connects innate and adaptive immunity. Different viruses trigger distinct forms of ICD, including necroptosis, pyroptosis, and autophagy, which amplify the release of PAMPs, DAMPs, and type I interferons. Cytosolic viral nucleic acids simultaneously activate cGAS–STING and RIG-I pathways, producing interferon-stimulated genes that attract and mature BATF3^+^ dendritic cells within the tumor microenvironment. These activated dendritic cells cross-present tumor antigens to CD8^+^ T cells, broadening the T-cell receptor repertoire and promoting durable systemic antitumor immunity. The same type I IFN and ICD-related signaling cascades can also increase checkpoint molecules such as PD-L1, providing a biological explanation for the consistent synergy observed when oncolytic viruses are combined with PD-1 or PD-L1 blockade in preclinical and early clinical colorectal cancer models [14]. Functionally, oncolytic viruses operate as in situ, antigen-agnostic tumor vaccines, expanding intratumoral CD8^+^ T-cell infiltration and broadening epitope specificity [15]. This transformation converts immune “cold” MSS tumors into “hot” immunologically active microenvironments [13,16]. Multiple clinical-stage platforms have demonstrated cold-to-hot conversion, characterized by increased intratumoral T-cell infiltration and heightened responsiveness to checkpoint blockade [17]. Many engineered OVs are equipped with transgenes encoding cytokines such as GM-CSF, IL-12, or interferon-β, which further amplify local immune activation [13,18].

Beyond direct cytotoxicity and immune activation, OVs also target tumor vasculature and angiogenesis (Figure 1), which are critical drivers of CRC progression. Tumor growth relies on the formation of new blood vessels. OVs can infect and lyse tumor-associated endothelial cells, destroying the tumor’s vasculature and inducing localized hypoxia. Several OVs have also been shown to directly target angiogenic signaling by downregulating VEGF and disrupting the structural integrity of tumor vasculature. In colorectal cancer and other models, adenoviral and vaccinia backbones armed with soluble VEGF receptor inhibitors or anti-VEGF antibodies reduced endothelial proliferation, vessel permeability, and tumor perfusion, thereby limiting neovascularization and metastatic potential [19]. Additionally, armed vectors encoding endogenous angiogenesis inhibitors such as angiostatin or endostatin demonstrate in vivo anti-angiogenic activity through VEGF-independent suppression of endothelial proliferation and migration [19]. Both strategies strengthen the anti-angiogenic profile of OVs and complement their lytic activity within the tumor microenvironment, counteracting the central role of VEGF-driven angiogenesis in CRC and extending beyond the effects of conventional anti-VEGF drugs already used in clinical practice [19].

It is important to note that these mechanisms do not operate independently; when present together, they act in parallel and influence each other. Direct oncolysis initiates immune activation by releasing tumor antigens and danger signals, while suppression of VEGF improves vascular structure and allows deeper T-cell infiltration and activation within the tumor. Immunogenic cell death from viral infection further amplifies anti-angiogenic effects and contributes to broader remodeling of the tumor microenvironment [20].

The clinical development of OVs further demonstrates their relevance to CRC. Talimogene laherparepvec (T-VEC), the prototype FDA-approved OV, confers durable melanoma responses and is being deployed in increasingly rational combination strategies in solid tumors [21]. Additional viruses, including adenoviruses, vaccinia virus, measles virus, and reovirus, are being investigated, with some early-phase clinical trials now enrolling CRC patients [22]. Specifically, in metastatic CRC, Pexa-Vec (JX-594) and oncolytic adenoviral platforms have entered OV–ICI combination studies, with early data indicating feasibility and immune activation in treatment-refractory disease [23].

Despite increasing interest in OVs, significant knowledge gaps persist, underscoring the need for further investigation. These gaps span both fundamental and translational challenges, including limited understanding of how OVs behave within the heterogeneous CRC tumor microenvironment, uncertainty regarding optimal viral dosing and delivery, and a lack of standardized preclinical models that reliably predict clinical responses. This review analyzes the three principal mechanisms of oncolytic virotherapy in colorectal cancer: direct oncolysis, immune activation, and anti-angiogenic targeting. It evaluates these mechanisms as standalone treatments, in combination regimens, and with enhancer strategies. The aim is to outline current progress, identify ongoing challenges, and assess the potential of OVs to address unmet needs in treatment-resistant CRC.

## 2. Materials and Methods

A comprehensive literature search was conducted between April and June 2025 using the PubMed database. The main objective was to identify primary studies investigating oncolytic viruses in colorectal cancer. The search strategy combined free-text terms and, where available, Medical Subject Headings (MeSH) to maximize sensitivity and specificity. The following keywords were utilized to develop the search strategy: oncolytic virus, colorectal cancer, and therapy. The exact PubMed search string applied was: (“Oncolytic Virus*” OR “Oncolytic Viral”) AND “Colorectal Cancer” AND Therapy NOT review. No filters were applied, thereby not restricting the study design, publication date, or species. The search retrieved 126 papers, of which 101 studies met the inclusion criteria after screening the titles, abstracts, and full texts.

Inclusion criteria for study selection were: (1) studies focused on colorectal cancer, including human cell lines, murine models, or patients with CRC; (2) investigations that focus on oncolytic viruses administered as standalone therapy, in combination, or with enhancer strategies; (3) preclinical, published, and in-progress clinical studies reporting therapeutic effects. Eligible outcomes included at least one efficacy or mechanistic readout (e.g., tumor growth inhibition, survival, response rate, immune activation biomarkers, viral replication/trafficking, or angiogenesis-related endpoints).

Exclusion criteria included: (1) studies not focusing on colorectal cancer; (2) non-primary papers, such as reviews or editorials; (3) studies in which the oncolytic virus was not the primary focus or was only used as an experimental tool; (4) articles published in a language other than English when a full-text English translation was unavailable. Conference abstracts without sufficient methodological detail, commentary pieces, and protocols without primary data were also excluded.

Screening and data extraction were performed independently by three reviewers using a pre-piloted, standardized workflow, with disagreements resolved by consensus. The extracted data were compiled into structured tables. They included: virus family, virus name, study type, model or cell type, mechanisms of action (direct oncolysis, immune activation, anti-angiogenesis, or other), combination therapy, synergistic effects, enhancer used, and enhancer type. For clinical trials, additional predefined variables were extracted, including trial phase, trial status, patient population, delivery route, adverse events, outcomes/endpoints, and quantitative information.

## 3. Results

From an extensive PubMed search, 126 studies were identified, of which 101 were extracted. Most studies were preclinical, with few significant clinical studies. Results are presented in three categories: standalone oncolytic viruses (Table 1), OVs combined with other therapies (primarily immunotherapies; Table 2), and OVs with an enhancer (Table 3). Across the studies, three primary mechanisms were of interest: direct oncolysis, immune activation, and angiogenesis targeting.

### 3.1. Standalone Oncolytic Viruses

When used as monotherapies in colorectal cancer, oncolytic viruses primarily act via direct oncolysis, exhibiting tumor-selective lytic activity in xenograft models and CRC cell lines. Immune activation and angiogenesis targeting, though less frequently reported, are noteworthy in some instances. Studies reporting standalone OVs as a possible treatment for CRC are depicted in Table 1.

For example, two recombinant adenoviruses, Ad312-E1A and Ad315-E1A, induced replication, lysis, and apoptosis in tumor cells with IGF2 loss of imprinting, an epigenetic modification present in CRC. The high selectivity of the viruses is seen in their ability to distinguish LOI-positive tumor cells from normal tissue [24,25]. Similarly, a modified Measles virus (rMV-SLAMblind) employed an alternative strategy, targeting Nectin-4 for selective portal entry and lysis of CRC cells [32]. Another example is a vesicular stomatitis virus (M51R/ΔM51) that induced apoptosis by disabling interferon production and disrupting Rae1-Nup98 in CRC cells [43].

Additionally, several other oncolytic viruses, including Herpes simplex virus type-1 (G207) and oncolytic vaccinia virus GLV-1h68 demonstrated direct lysis in CRC models [31,39]. The different OVs employed distinct mechanisms that aligned with their viral biology, highlighting the variability and efficacy of OV monotherapy in CRC.

Immune activation was not often the focus of monotherapy, but it did occur in several instances. For example, ORFV NA1/11 induced lytic and apoptotic activity and modulated cytokines by upregulating IL-7, IL-13, and IL-15, while downregulating IFN-γ receptor and CXCL16 [38]. Reovirus T3D also activated natural killer cells via Type I interferon pathways [41]. In the ex vivo model, OV-loaded mononuclear cells selectively targeted CRC liver metastases, sparing healthy hepatocytes.

In a clinical study, administration of reovirus to patients with KRAS-mutant metastatic CRC demonstrated oncolytic activity and immune stimulation. The treatment increased levels of IFN-γ, CD8^+^ T cells, and antigen-presenting cells, while downregulating miR-29a-3p, a microRNA known to suppress the tumor suppressor WWOX in CRC and promote tumor progression [48].

Angiogenesis targeting was also rarely reported in monotherapy studies; however, the ORFV strain NA1/11 reduced VEGF-B expression, resulting in decreased tumor vascularization in vivo [38]. The same clinical study, which also mentioned using Reovirus, pointed to a reduction in VEGF, IL-8, and other pro-angiogenic factors [48].

### 3.2. Oncolytic Virus Combination Therapies

**Figure 2:**
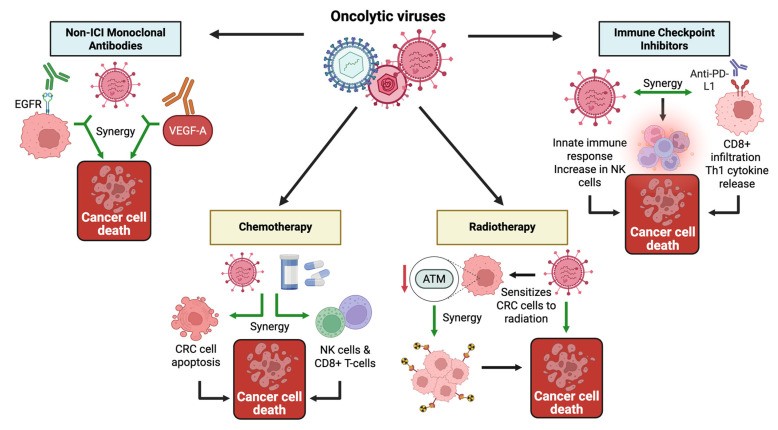
Oncolytic viruses in combination with other therapies to augment cancer cell death. Several synergistic methods pair up OVs with therapies such as immune checkpoint inhibitors, monoclonal antibodies, and other conventional therapies, including chemotherapy and radiotherapy, all of which increase cancer cell death directly or indirectly. Synergistic interactions depicted are based on preclinical evidence and will require confirmation in clinical studies. Created in BioRender. Busselberg, D. (2025) https://BioRender.com/mldkrjy. Abbreviations: ATM: Ataxia-Telangiectasia Mutated, CD8+ T cells: Cytotoxic T lymphocytes, CRC: Colorectal Cancer, EGFR: Epidermal Growth Factor Receptor, NK cell, Natural Killer cell; OV, Oncolytic Virus; PD-L1, Programmed death-ligand 1; Th1, Type 1 T helper cells; VEGF-A, Vascular Endothelial Growth Factor A. **Green** arrow: in synergy, **↓**: decreased.

Numerous studies, represented in Table 2, have investigated the use of OVs in combination with other therapeutic agents, particularly immunotherapies. These combinations aim to enhance immune activation, improve cytolytic activity, and, overall, overcome the resistance of CRC tumors. Notable synergy was most consistent in combinations involving immune checkpoint inhibitors, cytokine-enhanced viruses, and monoclonal antibodies. Other combinations, such as chemotherapy and radiation, also demonstrated benefits. Figure 2 illustrates the shared endpoint benefit of increased cancer cell death in the different combinations.

#### 3.2.1. Combinations with Immunotherapy

##### Immune Checkpoint Inhibitors

A significant number of studies have combined OVs with immune checkpoint inhibitors (ICIs), such as PD-1/PD-L1 inhibitors, which have consistently shown promising results. In one study, V937 (Coxsackievirus A21) combined with Pembrolizumab (PD-1 inhibitor) activated both innate and adaptive immune responses by increasing cytokines in CRC cell lines and organoids co-cultured with PBMCs [68]. Another PD-1 inhibitor, Nivolumab, was combined with the OV Enadenotucirev, which induced CD8^+^ T cell infiltration, increased Th1 cytokines, and synergistically prolonged overall survival in human CRC patients [81]. Additionally, the combination of anti-PD-1 and CSF-1R inhibition (PLX3397) with an adenovirus, Ad5-hTERT-E1A, increased tumor control in CRC mouse models [51].

CTLA-4 targeting has also been used in combination with OVs. To exemplify, in a phase I/II clinical trial by Monge et al. [23], Pexastimogene devacirepvec was combined with Tremelimumab (anti-CTLA-4) and Durvalumab (anti-PD-L1), which enhanced anti-tumor immune response by increasing tumor antigen release in patients with standard chemotherapy-refractory mismatch repair proficient (pMMR) metastatic colorectal cancer (mCRC). Moreover, Rojas et al. [74] investigated the combination of oncolytic vaccinia virus with anti-CTLA-4 and anti-CD25 antibodies in murine CRC models. They found increased NK and CD8^+^ T cells, along with decreased myeloid-derived suppressor cells (MDSCs). 

##### Monoclonal Antibodies (Other Than ICIs)

Monoclonal antibodies (mAbs) targeting angiogenesis and growth factor pathways have also been paired up with OVs. C-REV, an oncolytic herpes virus, was tested in combination with cetuximab in CRC cell lines and xenograft models. Pretreatment with Cetuximab before OV delivery significantly inhibited tumor growth by enhancing viral spread within the tumor and suppressing angiogenesis. The combination demonstrated synergy in vivo but not consistently in vitro [60].

Moreover, a Reovirus, Pelareorep, was investigated in a phase I clinical trial in KRAS-mutant metastatic CRC patients who were refractory or intolerant to oxaliplatin. The OV was combined with the anti-VEGF antibody bevacizumab and the standard chemotherapy regimen FOLFIRI. The study reported promising outcomes, including partial responses in 50% of patients who were treated with FOLFIRI for the first time, a median progression-free survival of 65.6 weeks, and an overall survival of 25.1 months. The combination allowed enhanced viral replication, rapid dendritic cell maturation, and a CD8^+^ T-cell activation, meaning a sustained immune response across the treatment cycles [87].

#### 3.2.2. Other Synergistic Combinations

##### Chemotherapy

Chemotherapeutic agents have also been combined with OVs to enhance cytotoxicity on tumor cells. A clinical case report combined low-dose capecitabine with an oncolytic adenovirus (H101) and demonstrated a complete response in lymph node metastasis, as well as 19 months of progression-free survival. This was in addition to NK cell activation and CD8^+^ T cell infiltration [53]. Similarly, Reovirus was combined with irinotecan, resulting in enhanced apoptosis through caspase activation and cell cycle arrest in murine CRC models [75].

##### Radiotherapy and Radiolabeled Strategies

Radiation therapy and radioisotope delivery were also investigated as synergistic combinations. A gene-modified adenovirus, Telomelysin (OBP-301), was able to sensitize CRC cells to ionizing radiation by downregulating ATM, thereby enhancing radiation-induced DNA damage [52]. A radio-virotherapy approach was used by introducing a vaccinia virus engineered to express somatostatin receptors to receptor-negative CRC tumors. This allowed the uptake of peptide-receptor radiotherapy (^177^Lu-DOTATOC), improving its accumulation and therapeutic efficacy by increasing tumor killing and prolonging survival in murine peritoneal carcinomatosis models [69].

##### Novel Combination Strategies

Novel combinations are emerging, in addition to chemotherapy and radiotherapy, which have been explored in several studies, including targeted agents and cell-based therapies. For example, a Reovirus was combined with the STAT3 inhibitor napabucasin, which enhanced apoptosis, induced cell cycle arrest, and downregulated KRAS and STAT3 signaling in CRC models. This highlights the potential of utilizing pathway-specific inhibitors to augment the efficacy of OVs [75]. Another study tested a Newcastle disease virus with Lactobacillus casei extract, which synergistically enhanced apoptosis and ROS production. This expanded the field to the possibility of incorporating agents derived from the microbiome [65]. Furthermore, some drugs have been repurposed and investigated with OVs. A study by Hu et al. [63] combined an oncolytic adenovirus with propranolol, demonstrating reduced VEGF secretion and tumor angiogenesis, resulting in improved tumor control. Another example by Li et al. [58] combined an adenovirus with NK cell therapy. Synergistic antitumor effects were observed, as the combination promoted NK cell proliferation and cytotoxicity while suppressing angiogenesis.

**Table 3:** (**a**). Oncolytic Virotherapy with Enhancers in Preclinical Studies. (**b**). Clinical Trials: Oncolytic Virotherapy with Enhancers.

(a)										
Virus Family	Virus	Study Type	Model/Cell Type	Direct Oncolysis	Immune Activation	Angio Targeting	Other Mechanism	Enhancer Used	Enhancer Type	Citation
Adenoviridae	OA@CuMnCs (H101 oncolytic Ad-5 with Cu/Mn shell)	Preclinical (in vitro and in vivo)	1 murine CRC cell line; CT26 syngeneic tumors in BALB/c mice	**✓**	**✓** (↓ PD-L1; ↑ IFN-γ, IL-1β, TNF-α; ↑ CD4^+^/CD8^+^ infiltration; ↑ DC maturation)	**✖**	STING activation; ICD	Copper–manganese biomineral coating	Immune evasion and hypoxia relief	[88]
	Oncolytic virus Ad·(ST13)·CEA·E1A(Δ24)	Preclinical (in vitro and in vivo)	4 human CRC cell lines; xenograft model in nude mice	**✓**	**✖**	**✖**	Mitochondrial apoptosis via caspase 9/3	Gene-armed OV (ST13 tumor suppressor)	Genetic engineering	[89]
	rAd.DCN.GM (decorin + GM-CSF)	Preclinical (in vitro and in vivo)	2 human CRC lines; CT26 syngeneic tumors in BALB/c mice	**✓**	**✓** (↑ CD8^+^ T cells, perforin/granzyme B; ↑ DCs)	**✓**	N/A	Decorin + GM-CSF gene arming	Cytokine arming/genetic engineering	[90]
	Wnt-targeted NTR-armed adenovirus	Preclinical (in vitro and in vivo)	4 human CRC lines; SW620 xenografts in NMRI nu/nu mice	**✓**	**✖**	**✓**	Bystander killing effect	Nitroreductase (NTR)	Tumor targeting/genetic engineering	[57]
	Oncolytic Adenovirus (CRAd5/F11)	Preclinical (in vitro and in vivo)	CRC cell lines; SW620 xenografts in BALB/c nude mice	**✓**	**✖**	**✓**	Tumor-specific tropism via MenSCs	Menstrual blood-derived mesenchymal stem cells (MenSCs)	Tumor targeting/immune evasion/enhanced delivery	[91]
	ZD55-VEGI-251	Preclinical (in vitro and in vivo)	1 human CRC line; SW620 xenografts in BALB/c nude mice	**✓**	**✖**	**✓** (VEGI-mediated antiangiogenesis)	mitochondria-mediated apoptosis via caspase-9/-3	Secreted isoform of VEGI (VEGI-251)	Genetic arming/tumor targeting	[92]
	dl1520 (ONYX-015)	Preclinical (In vitro)	9 Human CRC and normal cell lines	**✓**	**✖**	**✖**	↑ Tumor selectivity via differential viral replication at fever temp	Febrile temperature (39.5 °C)	Tumor targeting/Safety	[93]
	rAd.mDCN.mCD40L	Preclinical (in vitro and in vivo)	2 human CRC lines; 1 murine CRC line (CT26); CT26 murine CRC tumors in BALB/c mice	**✓**	**✓** (↑ CD8^+^, CD4^+^ memory, Th1 cytokines, ↓ Th2)	**✖**	Decorin inhibited TGF-β signaling → ↓ immune suppression; ↓ Met expression in CRC cells (anti-metastatic)	Decorin (mDCN) and CD40 Ligand (mCD40L)	Genetic engineering/Immune activator	[94]
	Oncolytic adenoviruses (ADVNE and ADVPPE)	Preclinical (in vitro and in vivo)	2 murine CRC lines; CT26 and MC38 tumors in C57BL/6 and BALB/c mice	**✓**	**✓** (Pyroptosis → HMGB1 release → TLR4 activation → ↑ M1 polarization)	**✖**	Tumor microenvironment modulation via macrophage reprogramming; overcoming T cell suppression	NE and PPE	Immune activators/Tumor targeting	[95]
	Ad5-D24-RGD	Preclinical (in vivo)	2 human CRC lines	**✓**	**✖**	**✓**	N/A	Macrophage Metalloelastase (MME)	Tumor Penetration	[96]
Herpesviridae	NV1042 (IL-12-secreting oncolytic HSV-1)	Preclinical (in vitro and in vivo)	1 murine CRC line; CT26 syngeneic flank tumors in BALB/c mice	**✓**	**✓** (IL-12 → ↑ T/NK cytotoxicity; IFN-γ induction)	**✖**	N/A	IL-12 cytokine arming	Localized immunomodulator/genetic engineering	[97]
	G207 (HSV with CEA-driven UL39)	Preclinical (in vitro and in vivo)	Human CRC lines; CRC xenografts in athymic nude mice	**✓**	**✖**	**✖**	N/A	CEA enhancer-promoter (CEA E-P)	Tumor targeting/Viral Replication	[98]
	Herpes simplex virus type 1 (VG22401)	Preclinical (in vivo and in vitro)	1 murine CRC line and 2 human CRC cell lines; CT26-HER2 syngeneic CRC model in Balb/c mice	**✓**	**✓** (anti-HER2 T cells and antibodies; ADCC, CDC; ↑ IFNγ^+^ splenocytes)	**✖**	N/A	Cytokine payload of IL-12, IL-15, and IL-15Ra	Localized immunomodulator/genetic engineering	[99]
	HSV1716	Preclinical (in vitro)	1 Human CRC cell line	**✓**	**✖**	**✖**	N/A	Ing4 (Inhibitor of Growth 4)	Virus replication/genetic engineering	[100]
	HSV-1	Preclinical (in vitro)	2 human CRC cell lines	**✓**	**✓** (↑ CD4+, CD8+ and macrophages; ↑ IFN-γ release and PBMC proliferation)	**✖**	N/A	IL-12 gene insertion	Localized immunomodulator/genetic engineering	[101]
	G207 (HSV-1)	Preclinical (in vitro and in vivo)	1 murine CRC line; CT26 tumors in BALB/c mice	**✓**	**✖**	**✖**	N/A	10xHRE upstream of UL39	Multimerized VEGF-derived hypoxia-responsive enhancer	[102]
	oncolytic SS2	Preclinical (in vivo and in vitro)	Human CRC line with CD133^+^ subpopulation; CRC xenografts in athymic nude mice	**✓**	**✖**	**✖**	N/A	CD133 promoter	Tumor targeting	[103]
Paramyxoviridae	NDV (rNDV-mOX40L)	Preclinical (In vitro and in vivo)	One murine CRC line; CT26 flank tumors in BALB/c mice	**✓**	**✓** (↑ CD4+, CD8+ and OX40+ T lymphocytes; ↑ IFN-γ and CTL activity)	**✖**	N/A	OX40L (OX40 ligand)	Immune activator	[104]
	NDV (S519G mutant)	Preclinical (in vitro and in vivo)	1 human CRC cell line; xenografts in nude mice	**✓**	**✖**	**✖**	N/A	S519G mutation in Hemagglutinin-Neuraminidase	Genetic engineering	[105]
Picornaviridae	Coxsackievirus B3 (Strain H3)	Preclinical (in vitro and vivo)	One human CRC line; DLD-1 xenografts in BALB/c nude mice	**✓**	**✖**	**✖**	N/A	miRNA-target site engineering (miR-375 and miR-1)	Tumor targeting	[106]
	Coxsackievirus A21 (CVA21)	Preclinical (in vivo)	murine colorectal cancer cells	**✓**	**✓** (↑ IFN-γ, ↓ IL-4, IL-10, TGF-β; ↑ splenocyte proliferation)	**✖**	N/A	Mesenchymal Stem Cells (MSCs)	Enhanced delivery	[107]
	CVB3 (Nancy, 31-1-93,H3, and PD)	Preclinical (in vitro and in vivo)	9 human CRC lines; DLD1 xenograft model	**✓**	**✖**	**✖**	N/A	N- and 6-O-sulfated Heparan Sulfate	Tumor Targeting	[108]
Poxviridae	VG9-IL-24 (IL-24-armed vaccinia)	Preclinical (in vitro and in vivo)	4 CRC cell lines; CT26 syngeneic tumors and HCT116 xenografts	**✓**	**✓** (↑ CTL activity, ↑ IFN-γ/IL-6/TNF-α, tumor-specific memory)	**✓**	G2/M arrest; apoptosis via PKR-p38/JNK; bystander effect	IL-24 cytokine arming	Localized immunomodulator/genetic engineering	[109]
	TPV/Δ2L/Δ66R/FliC (tanapoxvirus expressing flagellin)	Preclinical (in vitro and in vivo)	1 human CRC line; HCT116 xenografts	**✓**	**✓** (↑ lymphocyte and macrophage infiltration)	**✖**	FliC→TLR5-driven innate activation and necrosis	FliC (bacterial flagellin)	Innate immune activator (TLR5 agonist)	[110]
	vvDD-mIL2 (vaccinia expressing membrane-tethered IL-2)	Preclinical (in vivo; bilateral flank)	MC38-luc murine CRC line; MC38-luc syngeneic tumors in C57BL/6 mice	**✓**	**✓** (↑ CD8^+^ TILs; ↑ IFN-γ; ↑ CD11c^+^; abscopal immunity)	**✓**	N/A	Membrane-tethered IL-2	Localized immunomodulator/genetic engineering	[70]
	Vaccinia virus (VVLΔTKΔN1L-mIL-21)	Preclinical (in vitro and in vivo)	2 murine CRC cell lines; CMT93 and CT26 syngeneic models in C57BL/6 and BALB/c mice	**✓**	**✓** (CD8^+^ T cell killing; memory formation; ↑ IFN-γ; IL-21 modulation)	**✓** (IL-21 reduces VEGFR1 and TIE1 in endothelial cells)	N/A	Virus-encoded IL-21	Localized immunomodulator/genetic engineering	[111]
	vvTRAIL (TRAIL-armed vaccinia)	Preclinical (in vitro and in vivo)	2 human CRC lines; 1 murine CRC line; HCT116 and MC38 peritoneal carcinomatosis models in nude and C57BL/6 mice	**✓**	**✖**	**✖**	TRAIL→DR4/DR5 extrinsic apoptosis; ↑ cleaved caspase-8/PARP; bystander kill;	TRAIL gene arming	Death ligand/apoptosis inducer	[71]
	Oncolytic Vaccinia Virus-Luc@Ce6	Preclinical (in vitro and in vivo)	Human and murine colon cancer cell lines; CT26 syngeneic tumors in male BALB/c mice	**✓**	**✓** (↑ T cells, ↑ DCs, ↓ Tregs; induces ICD and boosts innate + adaptive immunity)	**✓**	N/A	Engineered to express firefly luciferase and surface-loaded with Chlorin e6.	Self-activating photodynamic enhancer	[112]
	PLTM-ICG-OVV (PIOVV)	Preclinical (in vitro and in vivo)	Murine colon cancer cell lines; syngeneic tumors in male BALB/c mice	**✓**	**✓** (Enhanced ROS accumulation)	**✓**	N/A	Indocyanine green (ICG), platelet membrane (PLTM) encapsulation	Photosensitizer/tumor targeting/enhanced delivery/immune evasion	[113]
	VVL15	Preclinical (in vivo)	CT26 murine colorectal cancer cell line	**✓**	**✓** (↓ Macrophage-mediated clearance)	**✖**	N/A	PI3Kδ inhibitor (IC87114 or Idelalisib	Immune evasion	[114]
	GLV-1h153	Preclinical (in vitro and in vivo)	2 human colorectal cancer cell lines; athymic nude mice (xenografts).	**✓**	**✖**	**✖**	N/A	hNIS (human sodium iodide symporter)	Enhanced Delivery and imaging enhancement	[115]
	Recombinant OVV	Preclinical (in vitro)	7 Human colorectal cancer cell lines	**✓**	**✖**	**✖**	N/A	lncRNA UCA1	Virus Spread	[116]
	Oncolytic Vaccinia Virus	Preclinical (in vitro and in vivo)	7 human and murine CRC cell lines; xenograft mouse models	**✓**	**✓** (↑ CD4^+^/CD8^+^; ↑ perforin and granzyme B cytotoxicity; ↑ cytokines)	**✖**	Bystander killing effect	Genetic modification to express bispecific T-cell engagers (TCEs)	Immune activation	[117]
	oncoVV-AVL	Preclinical (in vitro and in vivo)	2 human CRC cell lines; xenograft tumors in BALB/c nude mice	**✓**	**✖**	**✖**	N/A	AVL (marine C-type lectin)	Tumor targeting	[118]
Reoviridae	Oncolytic Reovirus	Preclinical (in vitro)	1 murine CRC cell line and mouse-derived MSCs; BALB/c mice	**✓**	**✖**	**✖**	↑ Apoptosis via RAS-caspase pathway; ↑ Secretome-mediated cytotoxicity	Infected MSC-derived secretome	Enhanced delivery	[119]
	Reovirus (ReoT3D)	Preclinical (in vitro)	1 murine CRC line; 1 murine fibroblast line	**✓**	**✖**	**✖**	↑ Apoptosis; ↑ Cell Cycle Arrest (G0/G1, G2/M)	Adipose-derived mesenchymal stem cells (AD-MSCs)	Enhanced delivery	[120]
	Reovirus (T3D)	Preclinical (in vitro)	1 murine CRC line; 1 murine fibroblast line	**✓**	**✖**	**✖**	↑ Apoptosis (Annexin V/PI flow cytometry)	low-intensity ultrasound	Enhanced delivery	[121]
	Reovirus type 3 Dearing strain (RC402)	Preclinical (in vitro)	2 human CRC cell lines	**✓**	**✖**	**✖**	N/A	Small extracellular vesicles (sEVs)	Systemic Immune activator	[122]
(**b**)										
**Virus Family**	**Virus**	**Trial Phase**	**Enhancer**	**Trial Status**	**Patient Population**	**Delivery Route**	**Adverse Events**	**Outcomes/Endpoints**	**Quantitative Information**	**Citation**
Herpesviridae	NV1020 (HSV-1)	Phase I	Genetic engineering	Completed	N = 12 mCRC liver-dominant	Single HAI NV1020	Mild fever/HA/rigors; transient LFT↑	Safety of HAI NV1020; day-28 tumor response	1 pt −39%, 1 pt −20%; 7 SD, 3 PD; strong hepatic clearance, minimal systemic virus	[123]

Abbreviations: AD-MSCs, Adipose-derived Mesenchymal Stem Cells; BHK-21, Baby Hamster Kidney-21; CPE, Cytopathic Effect; DF-1, Chicken Embryo Fibroblast Cell Line; DR4, Death Receptor 4; DR5, Death Receptor 5; FliC, Flagellin C; G2/M, Gap 2/Mitosis Phase; HN, Hemagglutinin–Neuraminidase; HS, Heparan Sulfate; ICG, Indocyanine Green; MenSCs, Menstrual Blood–Derived Stem Cells; MSCs, Mesenchymal Stem Cells; NDV, Newcastle Disease Virus; NO, Nitric Oxide; OVV, Oncolytic Vaccinia Virus; Ox, Oxaliplatin; PARP, Poly (ADP-Ribose) Polymerase; PLTM, Platelet Membrane; ROS, Reactive Oxygen Species; sEVs, Small Extracellular Vesicles; STING, Stimulator of Interferon Genes; TGF-β, Transforming Growth Factor Beta; TLR5, Toll-Like Receptor 5; TNF-α, Tumor Necrosis Factor Alpha; TNF-BP, Tumor Necrosis Factor–Binding Protein; TILs, Tumor-Infiltrating Lymphocytes; VEGFA, Vascular Endothelial Growth Factor A; VEGI, Vascular Endothelial Growth Inhibitor. **✓**, evidence for the mechanism reported by the authors; **✖**, not reported; ↑, increased; ↓, decreased; →, led to. Abbreviations: HAI, hepatic arterial infusion; HA, headache; LFT, liver function tests; SAE, serious adverse event.

### 3.3. Enhancers of Oncolytic Virus Therapy

**Figure 3:**
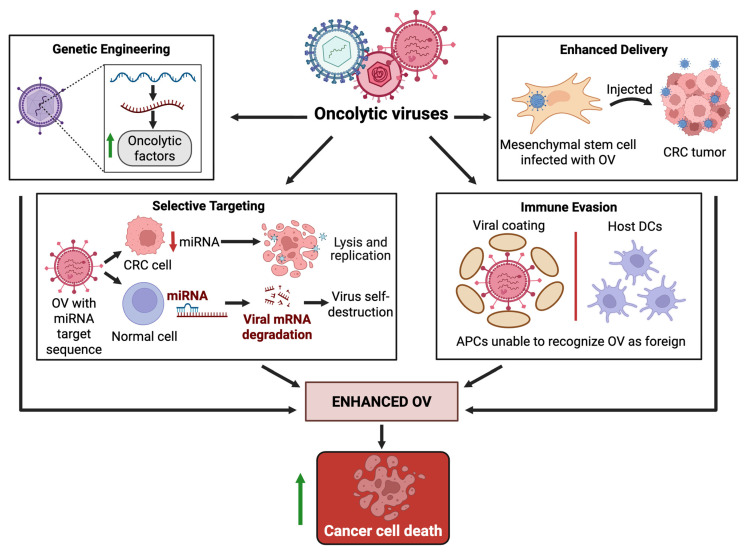
Four strategies of enhancement for oncolytic virotherapy. Enhancers aim to improve the potency and safety of OVs through four approaches: genetic engineering, delivery systems, selectivity in targeting, and immune evasion. Collectively, such strategies not only amplify therapeutic efficacy but also support the clinical translation of OVs. Created in BioRender. Busselberg, D. (2025) https://BioRender.com/nl69lr2. Abbreviations: APC, Antigen Presenting Cell; CRC, Colorectal Cancer; DC, Dendritic Cell; miRNA, microRNA; mRNA, messenger RNA; OV, Oncolytic Virus. **↑**: increased/enhanced, **↓**: decreased.

A wide range of strategies has been explored to enhance the delivery, selectivity, and therapeutic potential of OV in CRC. Table 3 presents the diverse range of enhancers investigated in combination with OVs for CRC therapy. The enhancers fall under functional categories that include, but are not limited to, increased delivery, oncolytic efficacy, selective tumor targeting, and immune evasion, all of which are illustrated in Figure 3, with the ultimate goal of enhancing CRC cell death.

Cell-based vehicles were often used to enhance OV delivery. Many studies have utilized mesenchymal stem cells (MSCs) as carriers, including adipose-derived MSCs with Reovirus and menstrual blood-derived MSCs with CRAd5/F11 adenovirus [72,91]. Low-intensity ultrasound was also tested as a means of increasing Reovirus penetration through ultrasound-induced sonoporation, which it successfully did [121].

Genetic and transcription modification methods were also employed to enhance tumor targeting and selectivity. Tumor-specific promoters were a recurring strategy, as evidenced by a study by Reinblatt et al. [98], which engineered CEA-secreting CRC cells to enhance HSV-1 (G207) replication and cytotoxicity, explicitly targeting the tumor. Hazini et al. [106] used miRNA-targeting strategies in Coxsackievirus B3 variants to restrict viral replication to tumor cells.

Additionally, OVs have been genetically modified to express immunostimulatory cytokines that stimulate an immune response, specifically in T cells. A vaccinia virus that encodes IL-21 (VVLΔTKΔN1L-mIL-21) increased CD8^+^ T cell-mediated killing, T-cell memory formation, and IFN-γ release. Additionally, it reduced the expression of VEGFR1 and TIE1 in endothelial cells, displaying the targeting of both angiogenesis and the immune response [111]. A recent study also engineered HER2-targeting HSV-1 to serve as a cytokine payload of IL-12, IL-15, and IL-15Ra. This resulted in T cell and humoral responses in the murine CRC models they used [99].

Photodynamic therapy (PDT) has also been studied to enhance the cytotoxic and immune-activating effects of OVs. One study used OVV-Luc@Ce6, a vaccinia virus engineered to express firefly luciferase and surface-loaded with Chlorin e6. This self-activating process induced localized phototoxicity within the cancer, which not only improved direct tumor cell killing but also induced immunogenic cell death, increased dendritic cells and T-cells, reduced T-regs, and overall enhanced innate and adaptive immunity [112].

Finally, angiogenesis targeting was also enhanced in several models. ZD55-VEGI-251 encoded a secreted isoform of VEGI that both inhibited angiogenesis and induced apoptosis [92].

## 4. Discussion

This review highlights the growing body of preclinical and early clinical studies on oncolytic viruses in colorectal cancer, emphasizing their diverse mechanisms and expanding therapeutic potential. Across the 101 included studies, direct oncolysis was universal (100%), immune activation was the most frequent secondary mechanism (44%), anti-angiogenic effects were reported in 16%, and all OV + ICI combinations evaluated demonstrated synergistic tumor control (100%). While the field appears promising, most data are preclinical, with only a few early-phase clinical studies in CRC patients, which is an explicit limitation. Accordingly, future CRC studies should incorporate delivery-optimization strategies (e.g., carrier cells, promoter/miRNA retargeting) and report standardized immunologic endpoints (CD8^+^ infiltration, antigen-presentation scores, interferon signatures) to enable cross-study synthesis [124].

Compared to melanoma, the only cancer in the US with an FDA-approved OV, T-VEC, CRC studies are still ongoing [125]. More recently, the FDA granted both breakthrough and fast-track designations in January 2024 to the oncolytic adenovirus Cretostimogene grenadenorepvec for non-muscle-invasive bladder cancer, reflecting one of the most advanced late-stage monotherapy OV programs currently in clinical development [126]. These advancements highlight the validated clinical potential for viroimmunotherapy, but CRC-specific efficacy data remain sparse [125]. Colorectal cancer is known for its immune-resistance and dense stromal environment, creating an obstacle to effective viral spread and immune involvement [127]. In these cold CRCs, OVs can initiate immune priming by inducing immunogenic cell death, which initiates the cancer-immunity cycle and drives dendritic cell activation and CD8^+^ T-cell trafficking into tumors [127]. PD-1/PD-L1 blockade appears to be the most frequently studied combination and is persistently effective. Preclinical CRC models and early clinical experiences show that OV + PD-1/PD-L1 increases intratumoral CD8^+^ T cells, Th1 cytokines, and tumor control, supporting cold-to-hot conversion [68,128]. This repeated success suggests that immune checkpoint inhibitors should be prioritized in regimens of future clinical trials. Notably, oncolytic adenovirus/vaccinia platforms paired with ICIs have entered CRC-relevant studies, underscoring feasibility and immunologic activity but also the need for harmonized endpoints and phase II/III confirmation [23,81].

In terms of immunotherapies, most studies have focused almost exclusively on checkpoint inhibitors. While this is currently promising, monoclonal antibodies beyond ICIs, such as those targeting EGFR or VEGF, are comparatively understudied in combination with OVs. Their established efficacy in CRC, combined with encouraging preliminary evidence when used in conjunction with OVs, warrants greater attention in the literature. Preclinical data already indicate that cetuximab + OV can yield synergistic tumor control in CRC models [60]. Early clinical experiences suggest that adding anti-VEGF to OV-containing regimens is feasible and may enhance intratumoral viral distribution [129]. Conversely, a randomized first-line study adding pelareorep (reovirus) to FOLFOX/bevacizumab did not improve progression-free survival, underscoring the need for biomarker-guided selection and smarter sequencing rather than blanket combination strategies [86].

It is worth mentioning that different enhancers work through distinct mechanisms, making them independent of one another. In other words, they should not be treated as competing strategies, but rather as complementary ones. Rational “stacking” of orthogonal enhancers—cell carriers + promoter/miRNA retargeting + immune-stimulatory payloads—is therefore justified, provided studies incorporate pharmacodynamic readouts (viral genomes, interferon signatures, CD8^+^ density) to detect synergy or antagonism [130].

By bringing together recurring significant findings across different viral families and CRC models, this review contributes to a growing understanding of how OVs may be rationally used, combined, and enhanced as a novel treatment for colorectal cancer. Compiling evidence demonstrating that OVs function as immune activators and tumor microenvironment remodelers presents a promising opportunity to address the current therapeutic gap. Researchers, clinicians, and institutions should use this information to develop CRC-specific virotherapy strategies, especially combinations that overcome CRC’s immunologically cold tumor characteristics. Priority areas include CRC-specific promoters (CEA/CDX2), VEGF/EGFR-axis-informed combinations, and delivery routes tailored to disease anatomy (intratumoral, hepatic-arterial, systemic), each paired with standardized endpoints to enable cross-trial comparisons [131].

### 4.1. Biomarkers for Patient Selection and Response Monitoring in OV Therapy

Biomarkers are increasingly essential to guide patient selection for oncolytic virotherapy in CRC. Many OVs rely on tumor-specific entry receptors or permissive antiviral states, making baseline tumor biology a key determinant of benefit [132]. For example, Coxsackievirus A21 (CVA21) requires intercellular adhesion molecule-1 (ICAM-1) as its cellular entry receptor; in prior models, tumor cell susceptibility to CVA21 was directly correlated with surface ICAM-1 expression, demonstrating that adequate receptor density is necessary for efficient viral attachment, internalization, and oncolysis [133]. Similarly, measles virus-derived OVs use nectin-4 (PVRL4), which is overexpressed in several adenocarcinomas [134]. These observations demonstrate that viral entry receptors can serve as predictive biomarkers; however, receptor expression alone is insufficient to predict responsiveness. A recent pancreatic cancer OV screen showed that tumor-intrinsic antiviral defenses strongly influence efficacy: high interferon-stimulated gene expression correlated with resistance, whereas impaired IFN signaling (e.g., low cGAS/STING activity) enhanced sensitivity [135]. Consistently, clinical studies report better OV responses in tumors with loss-of-function mutations in interferon pathway genes or other antiviral sensors [132]. Thus, permissiveness in CRC is shaped by both receptor availability and the absence of robust antiviral defenses.

Response-monitoring biomarkers further support real-time assessment of OV activity in CRC. Quantifying viral genomes or gene products in blood or tumor biopsies can confirm successful replication; biphasic viral DNA kinetics, for example, correlate with adequate intratumoral amplification and clinical benefit [136]. Immune-based pharmacodynamic markers—such as rises in IFN-γ, dendritic-cell activation, CD8^+^ T-cell recruitment, or induction of PD-L1—provide additional evidence that the OV is converting the CRC microenvironment toward an immune-active state [132]. Parallel monitoring of inflammatory markers (e.g., IL-6, CXCL9/10) is important for early detection of cytokine-mediated toxicity [137]. Together, predictive and pharmacodynamic biomarkers will be central to selecting CRC patients most likely to benefit from OVs and to verifying durable oncolysis and immune activation during therapy.

### 4.2. Practical Challenges of OV Delivery in Metastatic CRC

Systemic administration is typically required in metastatic CRC because multifocal liver and visceral lesions are inaccessible to intratumoral injection [138]. Yet intravenously delivered OVs are rapidly neutralized: virions are opsonized by IgM and complement, cleared by Kupffer cells and splenic macrophages, and inactivated by pre-existing antibodies and type I IFN responses, leaving only a small fraction available to reach tumors [138]. Within metastatic sites, the HA- and collagen-rich desmoplastic stroma increases interstitial pressure and physically limits OV spread. In contrast, hypoxic tumor regions constitute an additional barrier by suppressing viral replication and gene expression [127,138].

To overcome these systemic and tumor-intrinsic barriers, several enhancer strategies are under development. ECM-normalizing strategies, especially PH20 (hyaluronidase)-expressing vectors can degrade hyaluronan-rich stroma, improving intratumoral OV distribution and increasing CD8^+^ T-cell infiltration [127]. Additional strategies include cell-based carriers such as mesenchymal stem cells, which shield OVs from neutralizing antibodies and enhance tumor homing. Transcriptional retargeting platforms using CRC-specific promoters (including CEA and CDX2) or microRNA target sites to confine replication to malignant cells [98,106]. Together, these approaches aim to improve systemic OV bioavailability and enable more effective penetration of the stromal and hypoxic barriers characteristic of metastatic CRC.

### 4.3. Preclinical Modeling Barriers to Translating OV Therapy in CRC

The translational relevance of current CRC oncolytic virotherapy studies is constrained by reliance on subcutaneous xenografts, which fail to reconstruct the biological complexity of human colorectal tumors. These heterotopic models do not reproduce colon-specific extracellular matrix organization, crypt architecture, or stromal–epithelial structure, and they form non-physiologic vascular networks that distort tumor growth and therapeutic distribution [139]. Because subcutaneous tumors rarely metastasize, they also cannot model OV trafficking or immune modulation within relevant metastatic niches, such as the liver [139].

Immunologically, these systems poorly reflect the landscape of mismatch repair–proficient/microsatellite-stable (MSS) CRC. Subcutaneous xenografts lack colon-specific immune exclusion, altered cytokine gradients, and myeloid-dominant suppression [140]. Their use in immunodeficient hosts eliminates T-cell priming, NK-cell activity, antigen presentation, and antiviral interferon signaling, preventing meaningful evaluation of OV-induced immunogenic cell death or immune remodeling [141]. Stromal and vascular discrepancies further compound these limitations: human stroma is rapidly replaced by murine fibroblasts, altering angiogenesis, ECM density, cytokine signaling, and interstitial pressure, all of which influence viral penetration and replication [141]. Likewise, heterotopic vasculature fails to recreate the hypoxia gradients, vessel permeability, and angiogenic signaling characteristic of human CRC [139,140]. To bridge this translational gap, patient-derived organoids, orthotopic/CRLM models, and humanized-immune mouse systems should complement traditional subcutaneous xenografts, with aligned clinical-grade readouts (iRECIST, immune cell spatial profiling, circulating viral DNA) [130].

### 4.4. Safety and Toxicity Considerations

Across clinical trials, oncolytic viruses have demonstrated generally favorable safety profiles, with most adverse events limited to low-grade constitutional symptoms such as fever, chills, fatigue, nausea, and injection-site inflammation. A pooled analysis of 97 OV trials involving more than 3200 patients reported that severe treatment-related toxicities were uncommon and that high-grade immune-related adverse events occurred predominantly in OV–ICI combination studies rather than OV monotherapy [142]. Comparative evaluations across viral platforms further indicate that toxicity is largely driven by predictable antiviral immune responses, whereas organ-specific toxicities remain infrequent and manageable [143].

Viral shedding and off-target infection represent additional safety considerations. A recent systematic review found that shedding is commonly detectable by PCR but rarely involves replication-competent virus, and confirmed transmission to contacts or the environment is exceedingly rare [144]. Platform-specific characteristics also modulate safety, as some viruses elicit stronger cytokine responses or transient viremia [132]. Overall, current evidence supports a favorable therapeutic index for OVs, though systemic administration and combination regimens still require careful safety evaluation.

Despite ongoing challenges in delivery, modeling, and patient selection, the field of oncolytic virotherapy in CRC continues to advance, supported by emerging biomarker frameworks and strategies to overcome stromal and immunologic resistance. As these components improve, OVs may play a more meaningful role in treating MSS CRC, a population with limited therapeutic options. Continued integration of these innovations will be essential for translating preclinical promise into durable clinical benefit.

## 5. Conclusions

The field of virotherapy in colorectal cancer is advancing preclinically from standalone therapies to complex combinations and enhancer strategies. Monotherapies demonstrate direct oncolysis and immune activation, while combinations with immunotherapies, chemotherapy, radiotherapy, and novel strategies continue to expand the therapeutic potential. In vitro and in vivo experimental studies demonstrate OV’s potential to overcome the current problem of resistance in colorectal cancer that is refractory to various therapies, offering an opportunity when current treatments fail. The precision, safety, and efficacy of such therapy are continually improving, underscoring the need to translate preclinical findings into well-designed clinical studies. Despite current challenges and limitations, such as model heterogeneity and the need for standardization, the field is cautiously promising. Looking ahead, integrating oncolytic viruses with immune checkpoint inhibitors and enhancer strategies may represent the most effective path toward reshaping treatment outcomes in colorectal cancer.

Specifically, mismatch repair-proficient/microsatellite-stable (pMMR/MSS) colorectal cancer, classically referred to as “immune-cold,” represents the primary context in which oncolytic viruses can function as in situ vaccines, reprogramming the tumor microenvironment and restoring susceptibility to checkpoint blockade. Forthcoming CRC trials should incorporate biomarker-driven design, prospectively compare OV backbones, and predefine enhancer modules (carrier-cell delivery, tumor-specific promoters, miRNA de-targeting) alongside standardized immune and clinical endpoints to enable cross-study integration. Collectively, these features position OVs not as adjuncts but as backbone platforms for rational combination therapy, with the potential to lower the therapeutic ceiling in MSS disease and support eventual progression toward later-phase evaluation. As precision oncology advances, biomarker-based patient selection and adaptable OV platforms tailored to colorectal cancer profiles will be essential for achieving consistent therapeutic benefit. A sustained, collaborative effort to overcome biological, manufacturing, and delivery barriers will ultimately determine whether OV-based strategies can reach practical use in colorectal cancer.

## Figures and Tables

**Table 1:** (**a**). Oncolytic Viruses as Standalone Therapies in Preclinical Studies. (**b**). Clinical Trials: Oncolytic Viruses as Standalone Therapies.

(a)										
Virus Family	Virus		Study Type	Model/Cell Type		Direct Oncolysis	Immune Activation	Angio Targeting	Other Mechanism	Citation
Adenoviridae	Recombinant oncolytic adenovirus (Ad312-Early Region 1A)		Preclinical (in vitro and in vivo)	4 human cell lines (CRC + normal epithelial) + HT-29 xenograft mouse model		**✓**	**✖**	**✖**	N/A	[24]
	Ad315-E1A		Preclinical (in vitro and in vivo)	4 human CRC and normal epithelial cell lines; HCT-8 xenograft in athymic nude mice		**✓**	**✖**	**✖**	Apoptosis induction in Loss of Imprinting (LOI)+ cells	[25]
	EnAd-CMV-GFP		Preclinical (in vitro)	4 human CRC cell lines		**✓**	**✖**	**✖**	N/A	[26]
Herpesviridae	PRV (K61 and HB98 Strain)		Preclinical (in vitro)	1 human CRC cell line (HCT-8)		**✓**	**✖**	**✖**	Apoptosis via caspase-3 cleavage	[27]
	oHSV2 (Oncolytic Herpes Simplex Virus Type 2)		Preclinical (in vitro and in vivo)	2 human CRC cell lines; CT26 syngeneic CRC model in Bagg Albino (BALB/c) mice		**✓**	**✓** (GM-CSF → ↑ Dendritic cells, ↓ Tregs/Myeloid-Derived Suppressor Cells (MDSC), ↑ CD4^+^/CD8^+^ T cells)	**✖**	Necrosis (inflammatory cell death), cell cycle–independent killing	[28]
	oHSV2 (Oncolytic Herpes Simplex Virus Type 2)		Preclinical (in vivo)	CT26 syngeneic CRC model in BALB/c mice		**✓**	**✓** (T cells, B cells, Natural Killer cells, and neutrophils)	**✖**	N/A	[29]
	G207 & NV1020		Preclinical (in vitro and in vivo)	5 human CRC cell lines; xenograft model in male athymic mice		**✓**	**✖**	**✖**	N/A	[30]
	Herpes simplex virus type-1 (G207)		Preclinical (in vitro and in vivo)	5 human CRC cell lines; xenograft model in athymic rats; liver metastasis model in Buffalo rats		**✓**	**✖**	**✖**	Apoptosis via γ34.5 deletion	[31]
Paramyxoviridae (and Matonaviridae)	rMV-SLAMblind		Preclinical (in vitro and in vivo)	DLD1 and HumanTumor29 CRC xenograft models in SCID mice		**✓**	**✖**	**✖**	N/A	[32]
	PPRV M protein (matrix)		Preclinical (in vitro)	1 human CRC cell line		**✓**	**✖**	**✖**	BH3-like motif → Bax activation; intrinsic apoptosis	[33]
	Live-attenuated measles virus (Schwarz MV-eGFP)		Preclinical (in vitro and in vivo)	4 human CRC cell lines; Caco-2 xenograft model in nude mice		**✓**	**✖**	**✖**	CD46-mediated tumor selectivity; caspase-3-dependent apoptosis	[34]
	Newcastle disease virus AF2240 (velogenic)		Preclinical (in vitro)	7 human CRC cell lines (including p53 variant lines)		**✓**	**✖**	**✖**	N/A	[35]
	Measles, Mumps, Rubella (MMR)		Preclinical (in vivo)	Murine CRC		**✓**	**✓** TME remodeling, innate + adaptive activation	**✖**	N/A	[36]
Picornaviridae	Human enterovirus B species echovirus 12, 15, 17, 26 and 29		Preclinical (in vitro)	6 human CRC cell lines		**✓**	**✖**	**✖**	Apoptosis triggered by receptor binding (E12/E15, replication-independent)	[37]
Poxviridae	Orf Virus (ORFV) strain NA1/11		Preclinical (in vitro and in vivo)	7 CRC cell lines (human + murine); CT26 syngeneic model in Balb/c mice		**✓**	**✓** (↑ Interleukin-7, IL-13, IL-15, IL-21, CD27, CD30, CXCL13 → activation of T cells, NK cells, B cells)	↓ VEGF-B, ↓ Delta-like Ligand 4	Apoptosis (15 cytokines upregulated, CD27–SIVA pathway).	[38]
	oncolytic vaccinia virus GLV-1h68		Preclinical (in vitro and in vivo)	5 Human CRC cell lines; Xenograft in athymic rats		**✓**	**✓** (↑ IFN-γ, IP-10, MCP-1/3/5, RANTES, TNF-γ; ↑ macrophage and NK infiltration)	**✖**	IFN suppression; necrosis-driven immune influx	[39]
	CF33-Fluc		Preclinical (in vitro and in vivo)	3 human CRC cell lines; xenograft model in athymic nude mice		**✓**	**✓** (Necroptotic death; ↑ calreticulin, High Mobility Group Box 1 protein (HMGB1))	**✖**	Necroptosis	[40]
Reoviridae	Reovirus T3D		Preclinical (in vitro and ex vivo)	4 human CRC cell lines; human Peripheral Blood Mononuclear Cells (PBMC) and Liver Mononuclear Cells (LMC); 1 murine fibroblast line (L929)		**✓**	**✓** (NK activation via Type I IFN; PBMC and LMC degranulation and cytotoxicity)	**✖**	Innate immune effector activation in the liver	[41]
	Pelareorep		Preclinical (in vitro and in vivo, ex vivo PBMCs)	2 human CRC cell lines (KRAS mutant and WT isogenic pair); BALB/c and C57BL/6 mouse models		**✓**	**✖**	**✖**	Autophagy induction precedes apoptosis	[42]
Rhabdoviridae	Vesicular stomatitis virus M51R/ΔM51		Preclinical (In vitro)	1 human CRC cell line		**✓**	**✖**	**✖**	↑ Apoptosis; M-protein mutation disables interferon suppression	[43]
	Vesicular stomatitis virus (rwt and M51R mutant)		Preclinical (in vitro and in vivo)	3 human CRC cell lines; RKO and LoVo xenograft models in athymic nude mice		**✓**	**✖**	**✖**	Apoptosis induction	[44]
	Oncolytic vesicular stomatitis		Preclinical (in vitro and in vivo)	1 murine CRC cell line; 2 human CRC cell lines		**✓**	**✖**	**✖**	Syncytia formation and suicide gene strategies to enhance killing	[45]
(**b**)										
**Virus Family**	**Virus**	**Trial Phase**	**Trial Status**	**Patient Population**	**Delivery Route**	**Adverse Events**	**Outcomes/Endpoints**	**Quantitative Information**		**Citation**
Poxviridae	Pexastimogene devacirepvec JX-594	Phase Ib	Completed	N = 15 CRC-only (metastatic, refractory)	Intravenous infusion, biweekly	Mostly fever, chills, mild constitutional symptoms	Endpoint: safety + tumor response. Outcome: 10/15 had stable disease (SD); 5/15 had progression; no objective regressions	67 percent SD (10/15); 33 percent PD (5/15); no measurable shrinkage		[46]
	Pexa-Vec (oncolytic vaccinia virus)	Phase Ib	Completed	N = 6 Colorectal cancer liver metastases, neoadjuvant setting	Single 1 h intravenous infusion of 1 × 10^9^ pfu Pexa-Vec	Grade 3–4 lymphopenia/neutropenia	Endpoint: detect virus in tumor. Outcome: Virus found in 3/4 tumors; 2/6 showed major necrosis; 3/6 long-term cancer-free	Virus detected 3/4; major necrosis 2/6; long-term survivors 3/6		[47]
Reoviridae	Reovirus	Phase I	Completed	N = 5 KRAS-mutant metastatic CRC	IV infusion, 60 min/day × 5 days	No major safety findings reported	Endpoint: immune activation. Outcome: All showed APC and CD8 activation; miR-29a ↓; granzyme B ↑	Anti-tumor cytokines ↑; IL-8/RANTES ↓; granzyme B ~4× increase		[48]
	Reovirus	Clinical End-Point Trial	Completed	N = 10 metastatic CRC with liver metastases	Intravenous infusion of 10^10^ units reovirus	Fever/flu-like symptoms (6/10)	NK activation and IFN-I peak at 24–48 h, lost by 96 h; no re-activation with more doses	NK markers and ISGs peak 24–48 h; NK count ↑ up to 6–13×; no tumor response data		[49]

Abbreviations: BALB, Bagg Albino; DCs, Dendritic Cells; E1A, Early Region 1A; GM-CSF, Granulocyte–Macrophage Colony-Stimulating Factor; HCT, Human Colorectal Tumor; HMGB1, High Mobility Group Box 1 protein; HT, Human Tumor; IFN, Interferon; IL, Interleukin; KRAS, Kirsten Rat Sarcoma Viral Oncogene Homolog; LMC, Liver Mononuclear Cells; LOI, Loss of Imprinting; MCP, Monocyte Chemoattractant Protein; MDSCs, Myeloid-Derived Suppressor Cells; NK, Natural Killer; oHSV, Oncolytic Herpes Simplex Virus; ORFV, Orf Virus; PBMC, Peripheral Blood Mononuclear Cells; RANTES, Regulated upon Activation, Normal T-cell Expressed and Secreted; TNF, Tumor Necrosis Factor; Tregs, Regulatory T cells; VEGF-B Vascular Endothelial Growth Factor B. **✓**, evidence for the mechanism reported by the authors; **✖**, not reported; ↑, increased; ↓, decreased; →, led to. Abbreviations: SD, Stable Disease; PD, Progressive Disease; IV, Intravenous; pfu, Plaque-Forming Units; APC, Antigen Presenting Cell; IFN-γ, Interferon Gamma; IL, Interleukin; miR, MicroRNA; NK, Natural Killer; IFN-I, Type I Interferon; ISG, Interferon-Stimulated Gene.

**Table 2:** (**a**). Combining Oncolytic Viruses with Other Therapies in Preclinical Studies. (**b**). Clinical Trials: Combining Oncolytic Viruses with Other Therapies.

(a)										
Virus Family	Virus	Study Type	Model/Cell Type	Direct Oncolysis	Immune Activation	Angio Targeting	Other Mechanism	Combination Therapy	Synergistic Effect	Citation
Adenoviridae	CD55-TRAIL oncolytic adenovirus	Preclinical (in vitro and in vivo)	3 human CRC cell lines; mouse xenograft model	**✓**	**✖**	**✖**	Apoptosis	Luteolin	**✓**	[50]
	Type 5 Oncolytic Adenovirus (Ad5-hTERT-E1A)	Preclinical (in vivo)	2 murine CRC models (CT26, MC38) in BALB/c and C57BL/6 mice	**✓**	**✓** (↑ CD8^+^ T cells, ↑ IFNγ^+^ CD8^+^ T cells, ↑ ICOS)	**✓** (disrupted tumor neo-vasculature)	N/A	PLX3397 (CSF-1R inhibitor) + anti-PD-1	**✓** (↑ tumor control, ↑ survival; CT26: 43%, MC38: 82%)	[51]
	Telomelysin (OBP-301)	Preclinical (in vitro and in vivo)	1 human CRC cell line with PBMC and splenocyte coculture; HT29 rectal xenograft and lymph node metastasis mouse model	**✓**	**✖**	**✖**	Viral replication and trafficking suppressed lymph node metastasis	Ionizing radiation	**✓**	[52]
	H101 (Oncolytic Adenovirus)	Case Report	Patient with recurrent abdominal LN metastasis	**✓**	**✓** (↑ NK activation, ↑ CD8^+^ T cells, M1 polarization, ↓ MDSCs)	**✖**	N/A	Capecitabine (low-dose oral chemotherapy)	Complete response in 12 cm LN metastasis; Progression-Free Survival = 19 months; immune modulation	[53]
	Adv-CXCL10	Preclinical (In vitro and in vivo)	2 murine CRC cell lines; MC38 syngeneic mouse model	**✓** (only in vitro)	**✓** (T cell recruitment; ↑ IFN-γ and granzyme B in TME)	**✖**	Bystander effect, hypoxia induction	Anti-PD-1 antibody therapy	**✓** (by remodeling an antitumour immune microenvironment)	[54]
	ZD55-Dm-dNK	Preclinical (In vitro)	2 human CRC cell lines	**✓**	**✖**	**✖**	Suicide gene (Dm-dNK) activation of NA triggers selective apoptosis	Nucleoside analogs (BVDU, dFdC, ara-T)	**✓**	[55]
	Ad5/3-pCDX2 (CDX2-promoter oncolytic adenovirus)	Preclinical (in vitro and in vivo)	2 human CRC cell lines; subcutaneous xenografts and liver metastasis model in nude mice	**✓**	**✖**	**✖**	N/A	5-Fluorouracil (5-FU)	**✓**	[56]
	Wnt-targeted NTR-armed adenovirus	Preclinical (in vitro and in vivo)	4 human CRC cell lines; SW620 xenograft model in NMRI nu/nu mice	**✓**	**✖**	**✓**	Bystander killing effect	CB1954 prodrug + RAD001 (everolimus)	**✓**	[57]
	rAd.DCN	Preclinical (In vitro and in vivo)	2 human CRC cell lines; xenograft model in female NPG mice	**✓**	**✓** (↑ NK proliferation, infiltration, degranulation; ↑ perforin, IFN-γ)	**✓** (VEGF inhibition)	Decorin gene (delivered via rAd.DCN) targets TGF-β, Met and Wnt/β-catenin pathways	adoptive NK cell therapy	**✓**	[58]
	CRAdNTR	Preclinical (in vitro and in vivo)	3 p53-mutant human CRC cell lines; HT29 xenograft model in Balb/c mice	**✓**	**✖**	**✖**	Gene-Directed Enzyme Prodrug Therapy (GDEPT)	prodrug CB1954	**✓**	[59]
Herpesviridae	Oncolytic herpes virus C-REV	Preclinical (in vitro and in vivo)	3 human CRC cell lines; xenograft model in mice	**✓**	**✖**	**✓** (↓ VEGF, Basic Fibroblast Growth Factor (bFGF), and TGF-α)	N/A	Cetuximab	**✓** in vivo only	[60]
	oHSV (HSV-1 ∆810)	Preclinical (in vitro and in vivo)	1 murine CRC cell line; MC38 syngeneic CRC model in C57BL/6 mice	**✓**	**✓** (↑ CD8^+^/CD4^+^ infiltration; ↑ DC recruitment and activation)	**✖**	Immunogenic cell death and necroptosis	Low-dose mitomycin C + anti-PD-1 + anti-CTLA-4	**✓**	[61]
	oHSV2 (type II HSV-2 ICP47/ICP34.5-deleted)	Preclinical (in vitro and in vivo)	2 human DLBCL cell lines; 4 xenograft model in BALB/c mice	**✓**	**✓** (↑ CD4^+^/CD8^+^ infiltration; ↑ granzyme B and perforin)	**✖**	PD-L1 down-regulation; CTL activation	Anti-PD-L1 antibody	**✓** (strongest tumor inhibition; complete regression in 1/8 mice)	[62]
	T1012G	Preclinical (in vivo and in vitro)	3 human CRC cell lines; 2 murine CRC lines; xenograft models in BALB/c nude mice	**✓**	**✖**	↓ VEGF secretion; ↓ Tumor angiogenesis	N/A	propranolol	**✓**	[63]
Paramyxoviridae	Newcastle disease virus (LaSota)	Preclinical (in vitro)	1 human CRC cell line	**✓**	**✖**	**✖**	Intrinsic apoptosis (↑ caspase-9)	Bacillus coagulans ± 5-FU	**✓**	[64]
	Newcastle Disease Virus (NDV)	Preclinical (In vitro)	1 murine CRC cell line; MSCs from BALB/c mice	**✓**	**✓** (caspase-8/9 activation; LDH release; Th1, CTL, NK responses)	**✖**	↑ apoptosis via intrinsic and extrinsic pathways; ROS-mediated stress	Lactobacillus casei extract	**✓** (Enhanced apoptosis, ROS, and LDH levels vs. monotherapy)	[65]
Picornaviridae	Coxsackievirus A11 (CVA11)	Preclinical (in vitro and in vivo)	2 human CRC cell lines (oxaliplatin-sensitive and resistant); WiDr xenograft model in BALB/c nude mice	**✓**	**✖**	**✖**	N/A	Oxaliplatin	**✓** (↓ tumor volume & ↑ survival vs. monotherapy)	[66]
	Coxsackievirus B3 (PD-H)	Preclinical (in vitro)	1 refractory human CRC cell line	**✓**	**✖**	**✖**	N/A	FOLFOXIRI (oxaliplatin + SN-38 + 5-FU/folinic acid)	**✓** (synergistic cytotoxicity across all tested doses)	[67]
	V937 (Coxsackievirus A21)	Preclinical (in vitro)	CRC cell lines ± PBMCs; CRC organoids ± PBMCs	**✓** (ICAM-1+)	**✓** (with PBMC: ↑ cytokines; innate + adaptive responses)	**✖**	N/A	Pembrolizumab (PD-1 inhibitor)	**✓**	[68]
Poxviridae	Vaccinia virus (vvDD- Somatostatin Receptor)	Preclinical (in vivo)	CT26 murine CRC peritoneal carcinomatosis model in mice	**✓**	**✖**	**✖**	Bystander effects (radiation + OV danger signals)	^177^Lu-DOTATOC (peptide-receptor radiotherapy)	**✓**	[69]
	vvDD-mIL2 (oncolytic vaccinia virus)	Preclinical (in vivo)	MC38-luc syngeneic CRC model in C57BL/6 mice	**✓**	**✓** (↑ CD8^+^ T cells, ↑ TNF-α^+^ CD8^+^, ↑ IFN-γ^+^ CD4^+^, ↑ CD8^+^/Treg ratio)	**✖**	Membrane-tethered IL-2 delivers localized IL-2	CpG ODN (TLR9 agonist)	**✓** (slowed growth of contralateral tumors; ↑ median survival 27–33%)	[70]
	vvTRAIL (TRAIL-armed vaccinia)	Preclinical (in vitro and in vivo)	2 human CRC lines and 1 murine CRC line	**✓**	**✖**	**✖**	TRAIL-triggered apoptosis	Oxaliplatin	**✓**	[71]
	oncolytic poxvirus (vvDD-CXCL11)	Preclinical (in vitro and in vivo)	1 murine CRC line; MC38 peritoneal carcinomatosis model in C57BL/6 mice	**✓**	**✓**	**✖**	N/A	CKM (IFN-α, poly I:C, COX-2 inhibitor)	**✓**	[72]
	Oncolytic Vaccinia virus	Preclinical (in vitro and in vivo)	2 human CRC lines; 1 murine CRC line (MC38); xenografts in BALB/c nu/nu mice	**✓**	**✖**	**✖**	N/A	irinotecan (SN-38 in vitro; CPT-11 in vivo)	**✓** (Strong synergy in vitro; only DLD1 model translated to in vivo model synergy)	[73]
	Oncolytic Vaccinia virus	Preclinical (in vivo)	MC38 subcutaneous tumors in C57BL/6 mice	**✓**	**✓** (↑ NK cells, ↑ CD8^+^ T cells, ↓ MDSCs, ↑ antitumor responses, ↑ immune memory)	**✖**	N/A	anti-CTLA4 antibody, anti-CD25 antibody,	**✓**	[74]
Reoviridae	ReoT3D	Preclinical (In vitro)	1 murine CRC cell line	**✓**	**✖**	**✖**	↑ Apoptosis via caspase + STAT3 and KRAS downregulation	CPT-11 (irinotecan), BBI608 (napabucasin)	**✓** (↑ apoptosis, cell cycle arrest, gene regulation)	[75]
	Reovirus	Preclinical (In vitro)	KRAS mutant CRC cell lines	**✓**	**✖**	**✖**	↑Autophagy, ↑ Apoptosis	Carbamazepine	**✓** (combo > mono)	[76]
	Reovirus	Preclinical (in vitro and in vivo)	3 murine CRC cell lines; CT26 subcutaneous tumor model in BALB/c mice	**✓**	**✓** (↑ CD8+ T cell trafficking; ↑ IFN-β expression)	**✖**	N/A	STING agonist (ADU-S100)	**✓**	[77]
	Reovirus (ReoT3D)	Preclinical (In vitro)	1 murine CRC cell line	**✓**	**✖**	**✖**	Induction of apoptosis	Irinotecan + Metformin + MSC-derived ReoT3D secretome	**✓** (increased apoptosis and reduced cell viability)	[78]
	Reovirus T3D	Preclinical (in vivo)	C26-luc murine CRC liver metastases; BALB/c mice ± CyA	**✓**	**✖**	**✖**	N/A	Cyclosporin A (immunosuppressant)	**✓**	[79]
Rhabdoviridae	VSVΔ51	Preclinical (in vitro, in vivo, organoid)	Drug-resistant CRC lines (5 human, 1 murine); CRC organoids; HCT116/OXA (nude) and MC38/OXA (C57BL/6)	**✓**	**✓** (↑ NK infiltration, Granzyme B, IFN-γ, CD107a)	**✖**	Necroptosis	TBK1 inhibitor (GSK8612 or MRT67307)	**✓** (↑ viral replication and oncolysis; complete tumor regression in 1/5 mice)	[80]
(**b**)										
**Virus Family**	**Virus**	**Combination**	**Trial Phase**	**Trial Status**	**Patient Population**	**Delivery Route**	**Adverse Events**	**Outcomes/Endpoints**	**Quantitative Information**	**Citation**
Adenoviridae	Enadenotucirev	nivolumab	Phase I	Terminated Early	N = 51 45 CRC (mostly MSS/MSI-L), 6 SCCHN	IV enadenotucirev + IV nivolumab	61% grade 3–4; anemia, infusion reactions, hyponatremia, bowel obstruction; AKI/proteinuria signal	MTD not reached; minimal activity (ORR 2%, SD 45%); strong CD8^+^ immune activation	PFS 1.6 mo; OS 16 mo; 12-mo OS 69%; CD8 ↑ in 12/14	[81]
	Onyx-015	5-FU and leucovorin	Phase I/II	Completed	N = 24 Metastatic CRC	Hepatic artery infusion Onyx-015; later combined with IV 5-FU/LV	No DLTs; treatment well tolerated across ~200 infusions	PR 2/24; SD 11/24; transient tumor swelling before necrosis	Median OS 10.7 months; 1-year OS 46 percent; SD subgroup OS 19 months	[82]
Herpesviridae	NV1020 (Herpes Simplex Virus)	Intra-arterial chemotherapy (floxuridine ± irinotecan/oxaliplatin)	Phase I	Completed	N = 12 Metastatic CRC with liver-only disease	Hepatic arterial infusion (single dose)	No HSV-related toxicity; no viral reactivation	Early biologic effect before chemo; CEA decline; some tumor shrinkage; all patients had partial response once chemo began	CEA drop 13–74%; tumor shrinkage in 2/12 (up to 39%); median survival 25 months	[83]
	T-VEC	Atezolizumab	Phase Ib	Completed	N = 34 TNBC (N = 10); CRC (N = 24)	Intratumoral hepatic injection + IV atezolizumab	Grade ≥3 AEs: TNBC 70%, CRC 54%; DLTs: 0 in TNBC, 3 in CRC; 1 fatal AE (CRC)	Very limited activity; TNBC ORR 10%; CRC ORR 0%	TNBC: 1 PR (10%), PFS 5.4 months, OS 19.2 months; CRC: ORR* 0%, PFS 3 months, OS 3.8 months	[84]
Poxviridae	Oncolytic vaccinia virus (TG6002)	TG6002 (HSV-1 + FCU1) + oral 5-FC	Phase I	Completed	N = 15 Liver-dominant mCRC	Intrahepatic artery infusion of TG6002 + oral 5-FC	14/15 AEs; 5/15 grade 3; 1 DLT (grade 3 MI)	Safety achieved; no RECIST responses	Virus/FCU1 detected in 10/13 tumors; PFS 1.05 mo; OS 5.4 mo	[85]
	Pexastimogene devacirepvec (oncolytic vaccina virus)	Tremelimumab (anti–CTLA-4) + Durvalumab (anti–PD-L1)	Phase I/II	Completed	N = 34 pMMR metastatic CRC, chemo-refractory	IV PexaVec + IV ICIs	Fever/chills common; 3 immune-related toxicities (colitis, myositis); 1 hypotension → discontinuation	Safety/feasibility met; minimal antitumor activity	1 PR (51% shrinkage); SD in 4 pts; PFS 2.1–2.3 mo; OS 5.2–7.5 mo	[23]
Reoviridae	Pelareorep	FOLFOX6 + bevacizumab	Phase II	Completed	N = 103 Metastatic CRC	IV	More neutropenia, hypertension, proteinuria; more bevacizumab discontinuation	Pelareorep increased ORR but worsened PFS; OS unchanged	PFS 7 vs. 9 mo; ORR 53% vs. 35%; response duration 5 vs. 9 mo; OS 19.2 vs. 20.1 mo	[86]
	Pelareorep	FOLFIRI/Bevacizumab	Phase I	Completed	N = 36 KRAS-mutant mCRC	IV pelareorep + FOLFIRI ± bevacizumab	Neutropenia, anemia, diarrhea, fatigue; fever; bevacizumab-related proteinuria	High disease control; partial responses at highest dose	PR 3/6 at RPTD; overall PR 6/30; SD 22/30; PFS 65.6 wks (RPTD); OS 25.1 mo (RPTD)	[87]

Abbreviations: 5-FU, 5-Fluorouracil; bFGF, Basic Fibroblast Growth Factor; CD55, Complement Decay-Accelerating Factor; CpG ODN, Cytosine–Phosphate–Guanine Oligodeoxynucleotide; CSF-1R, Colony Stimulating Factor 1 Receptor; CTL, Cytotoxic T Lymphocyte; CTLA, Cytotoxic T-Lymphocyte Antigen; DLBCL, Diffuse Large B-Cell Lymphoma; E1A, Early Region 1A; EGFP, Enhanced Green Fluorescent Protein; GDEPT, Gene-Directed Enzyme Prodrug Therapy; ICAM, Intercellular Adhesion Molecule; ICOS, Inducible T-cell COStimulator; KRAS, Kirsten Rat Sarcoma Viral Oncogene Homolog; LDH, Lactate Dehydrogenase; LN, Lymph Node; MSCs, Mesenchymal Stem Cells; PD-1, Programmed Cell Death Protein 1; PFS, Progression-Free Survival; STAT3, Signal Transducer and Activator of Transcription 3; TGF-α, Transforming Growth Factor Alpha; Th1, T Helper Type 1; TLR9, Toll-Like Receptor 9; TRAIL, TNF-Related Apoptosis-Inducing Ligand. **✓**, evidence for the mechanism reported by the authors; **✖**, not reported; ↑, increased; ↓, decreased; →, led to. Abbreviations: SCCHN, Squamous cell carcinoma of head and neck; AEs, Adverse events; AKI, Acute kidney injury; ORR, Objective response rate; PFS, Progression-free survival; OS, Overall survival; MTD, Maximum tolerated dose; 5-FU/LV, 5-fluorouracil/leucovorin; DLT, Dose-limiting toxicity; PR, Partial response; TNBC, Triple negative breast cancer; ORR*, Overall response rate; PR, Partial response; 5-FC, 5-Fluorocytosine; MI, Myocardial infarction; RECIST, Response Evaluation Criteria in Solid Tumors; RPTD = recommended phase two dose.

## Data Availability

No new data were created or analyzed in this study.

## Glossary

Abscopal effect: Regression of distant, untreated lesions after local therapy; attributed to systemic immune activation. Angiogenesis: Formation of tumor vasculature; targeted by anti-VEGF agents and by OVs via endothelial infection or anti-angiogenic transgenes. Antigen cross-presentation: Display of exogenous tumor antigens on MHC-I by dendritic cells; primes CD8^+^ T-cell responses after OV-induced cell death. Antigen spread: Broadening of immune responses from initial antigens to additional tumor epitopes following immunogenic tumor destruction. cGAS–STING pathway: Cytosolic DNA-sensing axis inducing type I interferons; activated by OV infection to couple innate sensing with T-cell priming. CEA (carcinoembryonic antigen): CRC-associated glycoprotein; used as a tumor-specific promoter/enhancer to restrict OV replication. Colorectal liver metastases (CRLM): Hepatic metastases from CRC; relevant to intratumoral or hepatic-arterial OV delivery. Cytokine-armed virus: OV engineered to express immune stimulants (e.g., GM-CSF, IL-12, IFN-β) to amplify local antitumor immunity. DALY (disability-adjusted life year): Population burden metric (years of life lost + years lived with disability); substantial for CRC. DAMPs (damage-associated molecular patterns): Signals (e.g., calreticulin, ATP, HMGB1) released during OV-induced immunogenic cell death. dMMR/MSI-H: Mismatch repair–deficient, microsatellite-instability–high tumors; high neoantigen load and sensitivity to ICIs. EMT (epithelial–mesenchymal transition): Program enabling invasion, metastasis, and therapy resistance; contributes to immune exclusion in CRC. Early-onset CRC (EOCRC): CRC diagnosed before age 50; rising incidence associated with lifestyle and microbiome factors. FOLFOX/FOLFIRI/FOLFOXIRI: Standard CRC chemotherapy backbones: 5-FU/leucovorin + oxaliplatin; 5-FU/leucovorin + irinotecan; all three combined. Hepatic-arterial infusion (HAI): Regional hepatic-artery delivery; considered for OV administration to maximize liver tumor exposure. ICD (immunogenic cell death): Pro-inflammatory tumor cell death that elicits antigen presentation and T-cell priming; commonly triggered by OVs. Immune checkpoint inhibitors (ICIs): Antibodies blocking PD-1/PD-L1 or CTLA-4; restore T-cell function and synergize with OVs in MSS CRC. miRNA de-targeting: Inserting tumor-specific microRNA target sites into OV genomes to suppress replication in normal tissues and enhance tumor selectivity. MDSCs (myeloid-derived suppressor cells): Immunosuppressive myeloid cells enriched in MSS CRC; can be reduced/reprogrammed by OV combinations. MSS/pMMR (“immune-cold” CRC): Mismatch repair–proficient, microsatellite-stable tumors; low T-cell infiltration and poor ICI response, prime for OV “cold-to-hot” conversion. Oncolysis: Selective tumor cell lysis due to productive OV replication with release of progeny virions and antigens. Oncolytic virus (OV): Natural or engineered virus that preferentially replicates in malignant cells; mediates oncolysis, immune activation, and anti-angiogenesis. Organoids (patient-derived): 3D ex vivo cultures preserving CRC architecture and heterogeneity; used to evaluate OV and ICI responses. Photodynamic virotherapy: OVs combined with photosensitizers (e.g., Ce6/ICG); light activation generates ROS, augments ICD, and enhances antitumor immunity. PRRT (peptide receptor radionuclide therapy): Targeted radiotherapy using peptide–radionuclide conjugates; OVs can introduce SSTR to enable PRRT. Sonoporation: Low-intensity ultrasound–induced membrane permeabilization; increases OV entry and intratumoral penetration. Synergy metrics: Quantification of drug–OV interactions (Bliss independence, Loewe additivity, HSA) to define combination effects. Tumor microenvironment (TME): Tumor, stromal, vascular, and immune components; shape viral spread and immune response, and are remodeled by OVs. VEGF (vascular endothelial growth factor): Pro-angiogenic cytokine driving tumor vascularization; targeted by bevacizumab and by OVs armed with anti-angiogenic or VEGF-inhibitory genes.
